# Apparent Hypothalamic-Pituitary-Adrenal Axis Suppression *via* Reduction of Interleukin-6 by Glucocorticoid Therapy in Systemic Autoimmune Diseases

**DOI:** 10.1371/journal.pone.0167854

**Published:** 2016-12-08

**Authors:** Natsuki Fujio, Shotaro Masuoka, Kotaro Shikano, Natsuko Kusunoki, Toshihiro Nanki, Shinichi Kawai

**Affiliations:** Division of Rheumatology, Department of Internal Medicine, School of Medicine, Faculty of Medicine, Toho University, Ota-ku, Tokyo, Japan; Nippon Medical School, JAPAN

## Abstract

**Context:**

Suppression of the hypothalamic-pituitary-adrenal (HPA) axis is a serious complication of systemic glucocorticoid therapy.

**Objective:**

To clarify the influence of proinflammatory cytokines on the HPA axis after onset of glucocorticoid therapy in patients with systemic autoimmune diseases.

**Patients and Methods:**

Forty-eight glucocorticoid-naïve patients with systemic autoimmune diseases (28 women) who were starting prednisolone therapy according to our standard regimens were prospectively observed. Patients were classified into high-dose and low-dose groups depending on the dose of prednisolone administered as indicated for their diseases. Plasma adrenocorticotropic hormone (ACTH) and serum cortisol levels were measured by electrochemiluminescence immunoassay. The corticotropin-releasing hormone (CRH) test was performed at baseline and second and forth weeks after starting glucocorticoid therapy. The increased levels of ACTH (ΔACTH) and cortisol (Δcortisol) were investigated. Serum levels of 10 proinflammatory cytokines were measured simultaneously by a multi-spot assay system.

**Results:**

In the high-dose group, both basal and stimulated levels of ACTH and cortisol were significantly decreased by glucocorticoid therapy. In the low-dose group, basal ACTH and cortisol levels were also significantly decreased by glucocorticoid therapy, but ΔACTH and Δcortisol were unchanged. Among 10 cytokines, only interleukin (IL)-6 was significantly decreased by glucocorticoid therapy in both groups and was more closely correlated with cortisol than ACTH. Basal cortisol level was positively correlated with serum IL-6 level in all patients before glucocorticoid therapy.

**Conclusion:**

In patients with systemic autoimmune diseases, apparent suppression of cortisol during glucocorticoid therapy may be partly mediated by reduced production of IL-6.

## Introduction

Glucocorticoids are widely used to treat a variety of diseases, including systemic autoimmune diseases. Although glucocorticoids generally improve the clinical outcome, various side effects can be difficult to manage, including suppression of the hypothalamic-pituitary-adrenal (HPA) axis [1].

Corticotropin-releasing hormone (CRH) is secreted by the hypothalamus and stimulates secretion of adrenocorticotropic hormone (ACTH) from the anterior pituitary gland, which then stimulates the adrenal cortex to produce cortisol in a circadian and stress-related fashion. In addition, there are other regulators of adrenal cortisol production, such as inflammatory molecules, that may have a particularly important influence on the HPA axis in inflammatory disorders [2,3].

In fact, an influence of inflammation on the HPA axis has been reported in various autoimmune diseases. A rat model of inflammatory arthritis shows elevation of ACTH and corticosterone levels at the onset of arthritis [4], as does a mouse model of colitis at disease onset [5]. There have also been several reports about the HPA axis in human systemic autoimmune and inflammatory diseases. Demir *et al*. [6] reported that the cortisol level after low-dose dexamethasone suppression was positively correlated with acute phase reactants in patients with polymyalgia rheumatica (PMR), suggesting that inflammation might stimulate cortisol secretion. Similarly, Crofford *et al*. [7] reported that endogenous interleukin (IL)-6 may stimulate the secretion of ACTH and cortisol in patients with early untreated rheumatoid arthritis. These reports suggest that non-CRH and non-ACTH factors, such as proinflammatory cytokines, may influence the HPA axis in various inflammatory conditions.

In the present study, we prospectively performed the CRH test and measured 10 proinflammatory cytokines in untreated patients with systemic autoimmune disorders before and after initiation of glucocorticoid therapy. Our objective was to clarify the effect of endogenous proinflammatory cytokines on the HPA axis during the initial phase of glucocorticoid therapy.

## Patients and Methods

### Patients and study protocol

A total of 48 patients with systemic autoimmune diseases were recruited at Toho University Omori Hospital for a prospective observational study. Previously untreated patients who were starting prednisolone therapy at daily doses from 5 mg to 70 mg [mean daily dose: 30.1 ± 2.6 mg (SEM)] according to our standard regimens were eligible for enrollment. High- and low-dose regimens of glucocorticoid therapy in our study were defined as administration of ≥ 30 mg daily prednisolone for the patients with life or organ threatening manifestations and ≤ 20 mg daily prednisolone for not life or organ threatening manifestations, respectively, principally in accordance with the algorithm of glucocorticoid therapy in Harrison’s Principles of Internal Medicine [8]. The high-dose group (30–70 mg/day, 25 patients) included 5 patients with systemic lupus erythematosus (SLE), 8 patients with polymyositis/dermatomyositis (PM/DM), 7 patients with vasculitis syndrome, 2 patients with adult onset Still’s disease, 1 patient with mixed connective tissue disease, 1 patient with systemic sclerosis (SSc), and 1 patient with IgG4 related disease. The low-dose group (5–20 mg/day, 23 patients) comprised 14 patients with PMR, 5 patients with remitting seronegative symmetrical synovitis with edema (RS3PE) syndrome, 3 patients with rheumatoid arthritis, and 1 patient with SLE.

Collection of fasting morning blood sample and CRH stimulation test were performed on the previous day or the same day of initial glucocorticoid treatment and also at the second and fourth week after glucocorticoid therapy. Serum samples were immediately frozen at −80°C until assays were performed.

This study was carried out in accordance with the Declaration of Helsinki and Ethical Guidelines for Medical and Health Research Involving Human Subjects by Ministry of Education, Culture, Sports, Science and Technology and Ministry of Health, Labour and Welfare of the Japanese Government. This study was approved by the Ethics Committees of Toho University Omori Medical Center (approval number: 25–215). All patients gave written informed consent before enrollment in the study.

### CRH test

For the CRH test, a bolus intravenous injection of 100 μg of human corticorelin (hCRH, Mitsubishi Tanabe Pharma Corporation, Osaka, Japan) was administered in the morning after an overnight fast and before the usual morning dose of prednisolone. Blood was collected for measurement of plasma ACTH and serum cortisol levels before administration of CRH, as well as 30 and 60 minutes afterward.

### Measurement of hormones and proinflammatory cytokines

Plasma ACTH and serum cortisol levels were determined by electrochemiluminescence immunoassays (Roche Diagnostics, Basel, Switzerland). The basal plasma ACTH and serum cortisol concentrations and the increase of each hormone after CRH stimulation (ΔACTH and Δcortisol, respectively) were examined.

Serum levels of proinflammatory cytokines (interferon (IFN)-ɤ, IL-1β, IL-2, IL-4, IL-6, IL-8, IL-10, IL-12p70, IL-13, and tumor necrosis factor –alpha (TNF-α)) were measured in duplicate by an electrochemiluminescence immunoassay (Proinflammatory Panel 1 (human) Kit, Meso Scale Diagnostics, Rockville, MD) using the MESO QuickPlex SQ 120 (Meso Scale Diagnostics, Rockville, MD) according to the manufacturer’s instructions.

### Statistical analysis

Statistical analysis was performed with Prism ver. 5.02 software (GraphPad Software, San Diego, CA). Numerical data were expressed as the mean ± SD or the median with the interquartile range (IQR). Assessment of changes during glucocorticoid treatment was performed with the Friedman test followed by Dunnett’s multiple comparison test. To compare two groups, the Mann-Whitney *U* test was applied for numerical data and Fisher’s exact test was used for categorical data. Correlation analysis was conducted with the Spearman ranked correlation test for nonparametric variables. Linear regression analysis was performed for bivariate analysis. The level of significance was set at *P*<0.05.

## Results

### Profile of the subjects

Table 1 shows the demographic and clinical data of the high-dose and low-dose groups. The low-dose group had a significantly higher mean age compared with the high-dose group, but there were no significant differences of the sex ratio, body mass index, height, weight, basal ACTH level, and basal cortisol level between the two groups. There were also no significant differences of the baseline levels of IFN-ɤ, IL-1β, IL-2, IL-4, IL-8, IL-13, and TNF-α. However, IL-6 was significantly elevated in the low-dose group compared with the high-dose group, while the levels of IL-10 and IL-12p70 were significantly elevated in the high-dose group relative to the low-dose group. There was no significant difference of C-reactive protein (CRP) between the two groups. The high-dose group was predominantly composed of patients with SLE, PM/DM, and vasculitis syndrome, while the low-dose group mainly included patients with PMR and RS3PE syndrome.

**Table 1:** Demographic and clinical data of the high-dose and low-dose groups at baseline.

	High-dose group	Low-dose group	*P* value
	(n = 25)	(n = 23)	
Age (yr)	63.1 ± 16.3	76.1 ± 7.6	
	69 [48–76]*	77 [70–81]*	*P* = 0.00562
Number of men/women	9/16	11/12	
Body mass index (kg/m^2^)	21.1 ± 2.9	22.0 ± 3.5	
	20.8 [19.6–22.7]	22.6 [19.8–24.8]	*P* = 0.24346
Height (cm)	157.4 ± 9.04	155.3 ± 9.2	
	155.0 [152.0–162.0]	152.2 [149.2–161.7]	*P* = 0.38595
Weight (kg)	52.7 ± 10.9	54.9 ± 11.1	
	48.6 [46.6–59.4]	54.9 [48.4–61.0]	*P* = 0.45744
Basal ACTH (pg/ml)	18.3 ± 12.4	19.5 ± 9.9	
	16.2 [9.2–21.8]	17.5 [14.0–27.5]	*P* = 0.05895
Basal cortisol (μg/ml)	14.6 ± 6.8	18.4 ± 7.1	
	13.6 [8.6–19.4]	16.7 [15.5–23.1]	*P* = 0.45744
Proinflammatory cytokines			
IFN-ɤ (pg/mL)	94.5 ± 149.0	39.3 ± 47.1	
	34.3 [14.0–137.6]	14.3 [10.9–48.7]	*P* = 0.10522
IL-1β (pg/mL)	0.33 ± 0.43	0.17 ± 0.22	
	0.18 [<0.02–0.44]	0.14 [0.07–0.19]	*P* = 0.41726
IL-2 (pg/mL)	0.30 ± 0.32	0.22 ± 0.47	
	0.33 [<0.02–0.51]	0.07 [<0.02–0.19]	*P* = 0.12821
IL-4 (pg/mL)	0.030 ± 0.034	0.021 ± 0.024	
	0.022 [<0.001–0.039]	0.016 [0.001–0.032]	*P* = 0.37568
IL-6 (pg/mL)	19.51 ± 33.40	43.95 ± 64.05	
	6.58 [2.47–22.54]*	27.19 [10.41–51.98]*	*P* = 0.01366
IL-8 (pg/mL)	43.7 ± 53.4	34.8 ± 20.0	
	21.4 [14.1–38.4]	26.7 [19.9–43.9]	*P* = 0.39174
IL-10 (pg/mL)	1.96 ± 1.85	1.07 ± 1.59	
	1.26 [0.90–2.41]*	0.59 [0.40–0.91]*	*P* = 0.01019
IL-12p70 (pg/mL)	0.48 ± 0.92	0.14 ± 0.11	
	0.26 [0.09–0.49]*	0.15 [0.04–0.21]*	*P* = 0.02307
IL-13 (pg/mL)	2.44 ± 1.97	2.15 ± 2.32	
	2.73 [<0.12–3.80]	2.30 [<0.12–2.80]	*P* = 0.31785
TNF-α (pg/mL)	53.4 ± 145.1	11.3 ± 11.4	
	12.1 [7.03–22.1]	9.81 [5.41–11.98]	*P* = 0.14005
CRP (mg/dl)	4.68 ± 5.08	6.32 ± 4.94	
	2.0 [0.5–9.7]	6.1 [2.2–8.6]	*P* = 0.11905
Initial prednisolone dose (mg/day)	46.3 ± 10.6	13.2 ± 3.6	
	50 [40–50]*	15 [10–15]*	*P* = 0.00000
	(30-70mg)	(5-20mg)	

ACTH; Adrenocorticotropic hormone, IFN; interferon, IL; interleukin, TNF; tumor necrosis factor, CRP; C-reactive protein. Data are the mean ± SD and median [25th to 75th percentile]. The high-dose group included 5 patients with systemic lupus erythematosus, 8 patients with polymyositis/dermatomyositis, 7 patients with vasculitis syndrome, 2 patients with adult onset Still’s disease, 1 patient with mixed connective tissue disease, 1 patient with systemic sclerosis, and 1 patients with IgG4 related disease. The low-dose group included 14 patients with polymyalgia rheumatica, 5 patients with remitting seronegative symmetrical synovitis with edema syndrome, 3 patients with rheumatoid arthritis, and 1 patient with systemic lupus erythematosus. Lower limits of detection range of IL-1β, IL-2, IL-4, IL-12, and IL-13 were 0.024, 0.027, 0.001, 0.034 and 0.120 pg/ml, respectively. Values under the limitation were treated as 0 in analysis.

*, *P*<0.05 for high-dose group compared with low-dose group by the Mann-Whitney *U* test.

### Basal ACTH and cortisol levels

**Fig 1:**
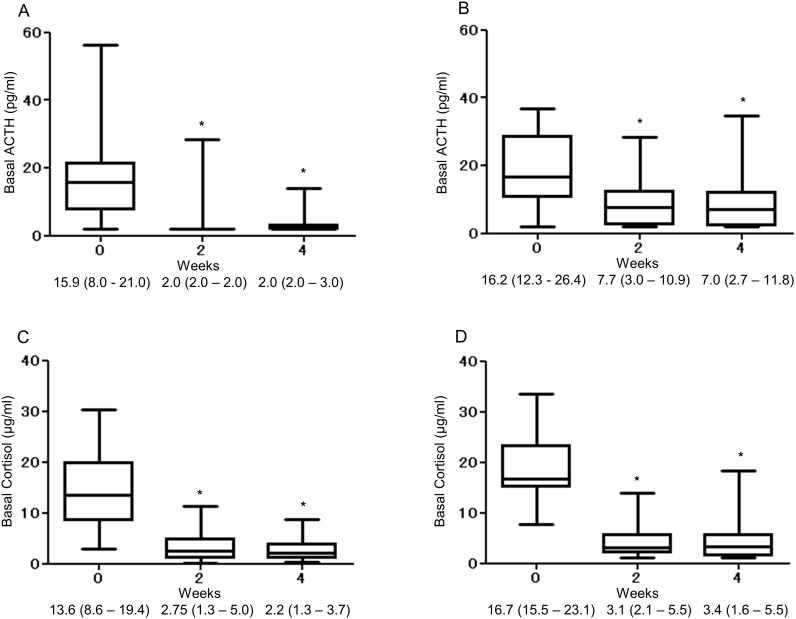
Changes of basal ACTH (A, B) and basal cortisol (C, D) during glucocorticoid therapy in the morning after an overnight fast in the high-dose group (A, C) and the low-dose group (B, D). Data are expressed as median values (25th to 75th percentiles). *, *P*<0.05 by Friedman’s test, followed by Dunnett’s multiple comparison test.

As shown in Fig 1(A) and 1(C), the basal ACTH and cortisol levels of the high-dose group decreased significantly after commencement of glucocorticoid therapy. Similarly, the basal ACTH and cortisol levels decreased significantly after the start of glucocorticoid therapy in the low-dose group (Fig 1B and 1D).

### CRH test

**Fig 2:**
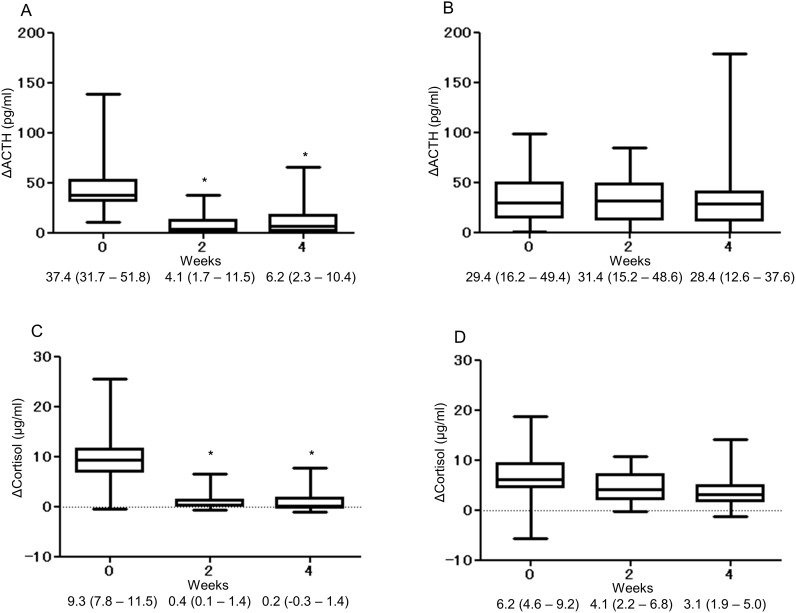
Response of ACTH and cortisol in the CRH test (ΔACTH (A, B) and Δcortisol (C, D), respectively) during glucocorticoid therapy in the high-dose group (A, C) and the low-dose group (B, D). Data are expressed as median values (25th to 75th percentiles). *, *P*<0.05 by Friedman’s test, followed by Dunnett’s multiple comparison test.

As shown in Fig 2(A) and 2(C), the ΔACTH and Δcortisol levels of the high-dose group decreased significantly after initiation of glucocorticoid therapy. In contrast, ΔACTH and Δcortisol did not decrease during glucocorticoid therapy in the low-dose group (Fig 2B and 2D).

### Proinflammatory cytokines

Data on the proinflammatory cytokines are displayed in Table 2. In the high-dose group, the serum levels of IFN-ɤ, IL-2, IL-4, and IL-8 were significantly decreased in the second week after commencing glucocorticoid therapy. Among the 10 proinflammatory cytokines, only IL-6 was significantly decreased in the second and fourth weeks of glucocorticoid therapy in both the high-dose and low-dose groups.

**Table 2:** Changes of serum proinflammatory cytokines.

	High-dose group (n = 25)			Low-dose group (n = 23)		
	Pre-GC therapy	after 2 weeks of GC therapy	after 4 weeks of GC therapy	Pre-GC therapy	after 2 weeks of GC therapy	after 4 weeks of GC therapy
IFN-ɤ	94.5 ± 149.0	34.1 ± 44.0	51.7 ± 61.3	39.3 ± 47.1	111.3 ± 330.2	190.6 ± 586.9
(pg/mL)	34.3 [14.0–137.6]	10.1 [6.7–39.6]*	17.9 [6.9–87.1]	14.3 [10.9–48.7]	14.6 [8.4–40.2]	25.1 [11.3–76.4]
IL-1β	0.33 ± 0.43	0.34 ± 1.04	0.13 ± 0.18	0.17 ± 0.22	0.19 ± 0.34	0.08 ± 0.11
(pg/mL)	0.18 [<0.02–0.44]	<0.02 [<0.02–0.15]	0.07 [<0.02–0.23]	0.14 [0.07–0.19]	0.04 [<0.02–0.25]	0.05 [<0.02–0.12]
IL-2	0.30 ± 0.32	0.14 ± 0.17	0.23 ± 0.32	0.22 ± 0.47	0.23 ± 0.51	2.67 ± 1.08
(pg/mL)	0.33 [<0.02–0.51]	0.05 [<0.02–0.24]*	0.15 [<0.02–0.29]	0.07 [<0.02–0.19]	0.03 [<0.02–0.17]	0.16 [<0.02–0.35]
IL-4	0.030 ± 0.034	0.012 ± 0.017	0.016 ± 0.017	0.021 ± 0.024	0.016 ± 0.026	0.009 ± 0.013
(pg/mL)	0.022	0.007	0.012	0.016	<0.001	<0.001
	[<0.001–0.039]	[<0.001–0.015]*	[<0.001–0.0214]	[0.001–0.032]	[<0.001–0.025]	[<0.001–0.013]
IL-6	19.51 ± 33.40	2.45 ± 2.54	3.21 ± 3.90	43.95 ± 64.05	5.48 ± 5.76	6.67 ± 7.71
(pg/mL)	6.58 [2.47–22.54]	1.35 [0.63–4.03]*	1.88 [0.83–4.03]*	27.19 [10.41–51.98]	4.01 [1.93–6.42]*	4.05 [2.31–7.43]*
IL-8	43.7 ± 53.4	26.2 ± 21.7	36.2 ± 34.5	34.8 ± 20.0	25.5 ± 15.2	31.3 ± 26.6
(pg/mL)	21.4 [14.1–38.4]	20.5 [10.6–28.7]*	24.7 [13.7–36.8]	26.7 [19.9–43.9]	22.2 [13.2–31.3]	25.8 [14.8–36.5]
IL-10	1.96 ± 1.85	2.73 ± 6.17	2.40 ± 3.86	1.07 ± 1.59	3.01 ± 9.02	5.93 ± 16.90
(pg/mL)	1.26 [0.90–2.41]	0.72 [0.38–1.93]	1.14 [0.50–2.54]	0.59 [0.40–0.91]	0.63 [0.34–0.83]	0.77 [0.43–1.23]
IL-12p70	0.48 ± 0.92	0.39 ± 0.99	0.41 ± 0.54	0.14 ± 0.11	0.14 ± 0.19	0.32 ± 0.48
(pg/mL)	0.26 [0.09–0.49]	0.17 [0.04–0.30]	0.26 [0.09–0.44]	0.15 [0.04–0.21]	0.10 [<0.03–0.19]	0.15 [0.06–0.24]
IL-13	2.44 ± 1.97	1.94 ± 2.25	2.12 ± 2.13	2.15 ± 2.32	1.57 ± 2.30	1.79 ± 2.06
(pg/mL)	2.73 [<0.12–3.80]	1.66 [<0.12–3.57]	2.18 [<0.12–3.09]	2.30 [<0.12–2.80]	<0.12 [<0.12–2.47]	1.41 [<0.12–2.61]
TNF-α	53.4 ± 145.1	13.9 ± 24.0	20.5 ± 50.5	11.3 ± 11.4	11.1 ± 13.7	34.6 ± 97.4
(pg/mL)	12.1 [7.03–22.1]	5.8 [3.8–10.7]	4.7 [2.6–7.6]	9.8 [5.4–12.0]	7.9 [4.8–11.7]	10.2 [4.8–15.0]

GC; Glucocorticoid, IFN; interferon, IL; interleukin, TNF; tumor necrosis factor. Data are the mean ± SD and median [25th to 75th percentile]. Lower limits of detection range of IL-1β, IL-2, IL-4, IL-12, and IL-13 were 0.024, 0.027, 0.001, 0.034 and 0.120 pg/ml, respectively. Values under the limitation were treated as 0 in analysis.

*, *P*<0.05 vs. basal value before GC therapy by Friedman’s test, followed by Dunnett’s multiple comparison test.

**Fig 3:**
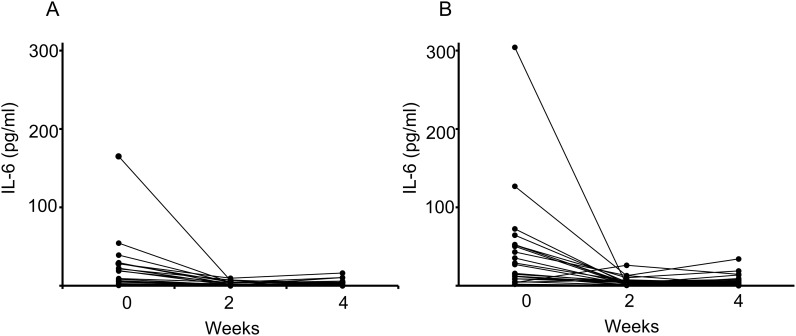
Individual changes of serum IL-6 from baseline after starting glucocorticoid therapy in the high-dose group (A) and the low-dose group (B).

Fig 3 shows individual changes of the serum IL-6 level from baseline. None of the patients had a high serum level of IL-6 after 2 weeks of glucocorticoid therapy in both the high-dose group (Fig 3A) and the low-dose group (Fig 3B).

To evaluate the influence of diseases on cytokine profiles, we compared changes of serum proinflammatory cytokines between patients with SLE, PM/DM and SSc, and those with vasculitis syndrome in the high-dose group (Table 3). We also compared changes of serum proinflammatory cytokines between patients with PMR and those with other diseases in the low-dose group (Table 4). Even after this categorization, only IL-6 among 10 proinflammatory cytokines was significantly changed during glucocorticoid therapy, with exception of SLE, PM/DM, and SSc patients at fourth week without a significant change, but a trend of reduction from baseline values (Table 3).

**Table 3:** Changes of serum proinflammatory cytokines between patients with SLE, PM/DM and SSc, and patients with vasculitis syndrome in the high-dose group.

High-dose group						
	SLE, PM/DM, SSc (n = 14)			Vasculitis syndrome (n = 7)		
	Pre-GC therapy	after 2 weeks of GC therapy	after 4 weeks of GC therapy	Pre-GC therapy	after 2 weeks of GC therapy	after 4 weeks of GC therapy
IFN-ɤ	115.3 ± 187.9	35.9 ± 45.8	53.9 ± 68.7	71.7 ± 88.1	33.1 ± 51.3	40.6 ± 52.1
(pg/mL)	36.9 [21.1–122.2]	11.7 [3.8–68.9]	16.2 [5.3–87.9]	32.4 [11.5–99.3]	6.9 [6.8–31.4]	17.9 [7.2–48.3]
IL-1β	0.37 ± 0.51	0.23 ± 0.47	0.14 ± 0.17	0.39 ± 0.38	0.75 ± 1.88	0.18 ± 0.23
(pg/mL)	0.14 [<0.02–0.58]	<0.02 [<0.02–0.25]	0.09 [<0.02–0.23]	0.29 [0.18–0.47]	<0.02 [<0.02–0.11]	0.10 [<0.02–0.26]
IL-2	0.32 ± 0.31	0.13 ± 0.15	0.18 ± 0.25	0.31 ± 0.39	0.20 ± 0.22	0.40 ± 0.47
(pg/mL)	0.34 [<0.02–0.54]	0.03 [<0.02–0.25]	0.04 [<0.02–0.27]	0.35 [<0.02–0.37]	0.17 [<0.02–0.29]	0.17 [0.08–0.62]
IL-4	0.027 ± 0.025	0.010 ± 0.012	0.022 ± 0.020	0.023 ± 0.026	0.023 ± 0.024	0.012 ± 0.009
(pg/mL)	0.027	0.007	0.018	0.015	0.014	0.015
	[0.004–0.038]	[<0.001–0.016]	[0.001–0.037]	[0.006–0.036]	[0.008–0.031]	[0.004–0.018]
IL-6	23.33 ± 43.42	2.70 ± 2.78	4.37 ± 4.80	21.02 ± 12.98	2.67 ± 2.75	2.02 ± 1.79
(pg/mL)	7.31 [1.95–21.63]	1.53 [0.71–4.20]*	2.71 [1.05–5.50]	21.68 [12.95–28.99]	1.84 [0.44–4.32]*	1.88 [0.69–3.21]*
IL-8	56.5 ± 68.7	26.8 ± 25.6	44.3 ± 42.7	28.2 ± 12.2	22.7 ± 13.8	24.1 ± 13.2
(pg/mL)	27.4 [12.9–77.6]	17.0 [9.5–27.0]	30.2 [14.5–54.8]	25.4 [20.7–33.1]	20.5 [15.8–25.8]	28.1 [13.3–30.4]
IL-10	1.77 ± 2.17	2.20 ± 4.66	2.24 ± 3.72	1.92 ± 1.07	4.93 ± 9.80	3.24 ± 5.10
(pg/mL)	1.06 [0.53–2.04]	0.64 [0.37–2.02]	1.25 [0.42–2.35]	1.78 [1.10–2.35]	1.53 [0.91–1.78]	1.42 [0.75–2.32]
IL-12p70	0.68 ± 1.20	0.54 ± 1.31	0.52 ± 0.67	0.22 ± 0.18	0.19 ± 0.18	0.23 ± 0.19
(pg/mL)	0.29 [0.20–0.61]	0.17 [0.09–0.28]	0.27 [0.11–0.44]	0.16 [0.08–0.22]	0.17 [0.08–0.22]	0.18 [0.10–0.40]
IL-13	2.63 ± 2.21	1.50 ± 2.05	1.63 ± 1.36	1.81 ± 1.30	2.62 ± 2.46	2.46 ± 2.18
(pg/mL)	3.06 [<0.12–4.39]	<0.12 [<0.12–2.71]	1.68 [0.15–3.04]	2.39 [0.89–2.73]	1.89 [0.83–4.03]	2.26 [1.09–3.25]
TNF-α	85.2 ± 190.7	20.3 ± 30.9	32.4 ± 66.0	11.3 ± 5.7	5.7 ± 2.3	6.9 ± 5.1
(pg/mL)	14.3 [5.75–27.2]	7.1 [3.2–16.3]	5.2 [2.6–15.6]	10.2 [9.3–12.6]	6.3 [3.8–7.0]	5.5 [4.5–6.53]

GC; Glucocorticoid, IFN; interferon, IL; interleukin, TNF; tumor necrosis factor, SLE; systemic lupus erythematosus, PM/DM; polymyositis/dermatomyositis, SSc; systemic sclerosis. Data are the mean ± SD and median [25th to 75th percentile]. Lower limits of detection range of IL-1β, IL-2, IL-4, and IL-13 were 0.024, 0.027, 0.001 and 0.120 pg/mg, respectively. Values under the limitation were treated as 0 in analysis.

*, P<0.05 vs. basal value before GC therapy by Friedman’s test, followed by Dunnett’s multiple comparison test.

**Table 4:** Changes of serum proinflammatory cytokines between patients with PMR and patients with other diseases in the low-dose group.

Low-dose group						
	PMR (n = 14)			Other diseases (n = 9)		
	Pre-GC therapy	after 2 weeks of GC therapy	after 4 weeks of GC therapy	Pre-GC therapy	after 2 weeks of GC therapy	after 4 weeks of GC therapy
IFN-ɤ	38.8 ± 47.6	20.5 ± 20.1	274.3 ± 746.3	40.1 ± 49.1	252.6 ± 512.2	60.3 ± 104.1
(pg/mL)	17.8 [10.8–35.4]	12.1 [7.1–24.9]	37.9 [15.5–77.5]	14.3 [13.6–59.4]	16.1 [13.1–134.1]	17.4 [7.4–64.4]
IL-1β	0.20 ± 0.28	0.14 ± 0.29	0.07 ± 0.07	0.12 ± 0.09	0.25 ± 0.41	0.11 ± 0.16
(pg/mL)	0.13 [0.07–0.21]	<0.02 [<0.02–0.15]	0.05 [<0.02–0.12]	0.14 [0.07–0.15]	0.10 [<0.02–0.27]	0.04 [<0.02–0.11]
IL-2	0.07 ± 0.08	0.08 ± 0.12	3.80 ± 13.6	0.46 ± 0.69	0.46 ± 0.76	0.61 ± 0.85
(pg/mL)	0.03 [<0.02–0.12]	0.01 [<0.02–0.12]	0.13 [<0.02–0.29]	0.11 [<0.02–0.49]	0.11 [<0.02–0.30]	0.20 [<0.02–1.07]
IL-4	0.015 ± 0.023	0.008 ± 0.024	0.006 ± 0.010	0.031 ± 0.024	0.027 ± 0.026	0.013 ± 0.016
(pg/mL)	0.008	<0.001	<0.001	0.035	0.027	0.012
	[<0.001–0.018]	[<0.001–<0.001]	[<0.001–0.008]	[0.016–0.051]	[0.005–0.048]	[<0.001–0.013]
IL-6	46.46 ± 77.28	5.40 ± 6.83	7.61 ± 8.88	40.05 ± 39.34	5.59 ± 3.94	5.20 ± 5.58
(pg/mL)	22.50 [9.63–48.56]	2.56 [1.52–5.84]*	4.31 [2.35–9.02]*	27.19 [12.24–52.01]	4.07 [3.72–7.56]*	4.07 [3.72–7.56]*
IL-8	32.6 ± 21.4	22.6 ± 13.7	36.1 ± 32.1	38.1 ± 18.2	30.1 ± 17.2	23.8 ± 12.9
(pg/mL)	23.4 [18.2–41.1]	19.8 [12.1–29.4]	29.1 [14.4–38.4]	36.7 [26.5–46.2]	22.7 [20.8–32.6]	25.8 [19.9–29.5]
IL-10	1.19 ± 1.93	3.54 ± 11.14	8.99 ±21.36	0.90 ± 0.91	2.19 ± 4.52	1.16 ± 1.49
(pg/mL)	0.62 [0.48–0.95]	0.61 [0.46–0.68]	0.80 [0.46–1.33]	0.51 [0.38–0.79]	0.76 [0.30–1.27]	0.77 [0.36–1.17]
IL-12p70	0.12 ± 0.12	0.07 ± 0.09	0.43 ± 0.60	0.18 ± 0.10	0.25 ± 0.24	0.15 ± 0.11
(pg/mL)	0.11 [0.10–0.21]	0.04 [<0.03–0.11]	0.13 [0.05–0.59]	0.18 [0.12–0.24]	0.20 [0.10–0.28]	0.15 [0.08–0.22]
IL-13	1.36 ± 1.38	0.50 ± 0.90	1.41 ± 1.55	3.37 ± 3.00	3.23 ± 2.84	2.38 ± 2.66
(pg/mL)	1.32 [<0.12–2.39]	<0.12 [<0.12–0.59]	1.26 [<0.12–2.17]	2.52 [2.09–3.82]	2.74 [2.15–3.16]	1.58 [<0.12–2.82]
TNF-α	8.2 ± 3.7	11.2 ± 17.0	50.6 ± 123.8	16.1 ± 17.0	10.9 ± 6.8	9.7 ± 5.4
(pg/mL)	9.1 [4.6–11.1]	7.2 [4.7–8.8]	7.6 [4.6–25.6]	11.0 [8.8–14.3]	10.5 [6.2–14.8]	10.2 [7.4–13.9]

GC; glucocorticoid, IFN; interferon, IL; interleukin, TNF; tumor necrosis factor, PMR; polymyalgia rheumatic. Data are the mean ± SD and median [25th to 75th percentile]. Lower limits of detection range of IL-1β, IL-2, IL-4, IL-12, and IL-13 were 0.024, 0.027, 0.001, 0.034 and 0.120 pg/mg, respectively. Values under the limitation were treated as 0 in analysis.

*, P<0.05 vs. basal value before GC therapy by Friedman’s test, followed by Dunnett’s multiple comparison test.

### Correlation between IL-6 and basal cortisol before glucocorticoid therapy

**Fig 4:**
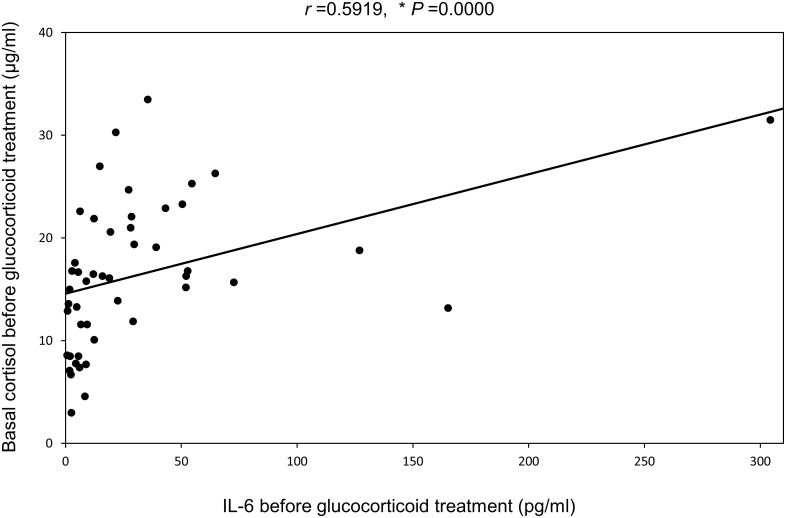
Correlation between IL-6 and basal cortisol before glucocorticoid therapy in patients with systemic autoimmune diseases. *, *P*<0.05 by Spearman’s rank correlation coefficient analysis.

Fig 4 shows correlation between levels of serum IL-6 and basal cortisol before glucocorticoid therapy. Levels of IL-6 and basal cortisol showed a significant positive correlation in all of the patients with systemic autoimmune diseases in this study.

### Relations among the changes of basal of ACTH, cortisol, and IL-6 levels

**Fig 5:**
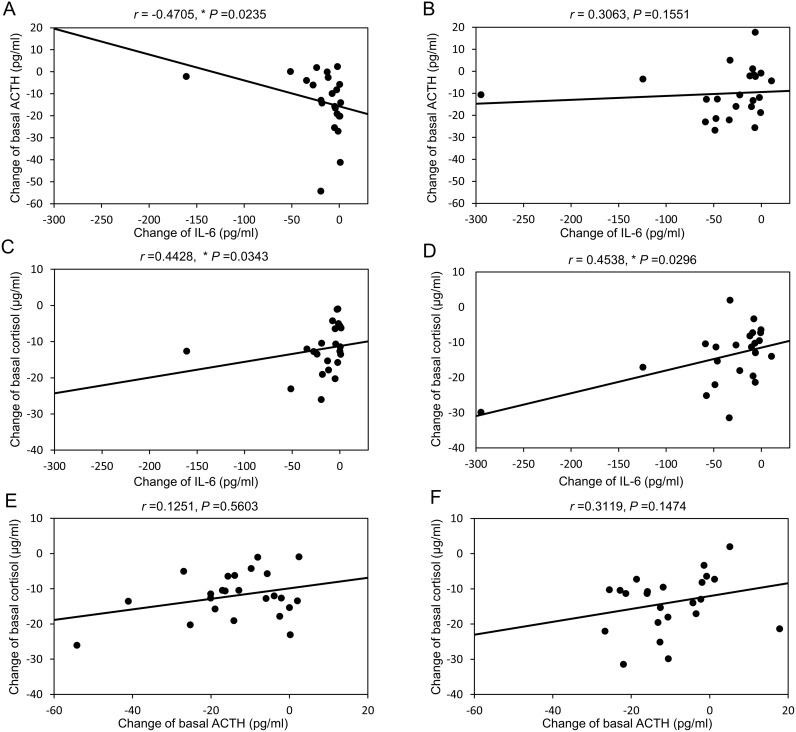
Correlations between the changes of IL-6 and basal ACTH (A, B), IL-6 and basal cortisol (C, D), and basal ACTH and basal cortisol (E, F) in the high-dose group (A, C, E) and the low-dose group (B, D, F). *, *P*<0.05 by Spearman’s rank correlation coefficient analysis.

During 4 weeks of high-dose glucocorticoid therapy, the change of basal ACTH showed a negative correlation with the change of serum IL-6 (Fig 5A), but a correlation was not found during low-dose glucocorticoid therapy (Fig 5B). The change of basal cortisol was positively correlated with the change of serum IL-6 during both high-dose (Fig 5C) and low-dose (Fig 5D) glucocorticoid therapy. In contrast, the change of basal ACTH was not significantly correlated with the change of basal cortisol during either high-dose (Fig 5E) or low-dose (Fig 5F) glucocorticoid therapy

## Discussion

In the present study, we investigated HPA axis function and proinflammatory cytokines before and after initiation of glucocorticoid therapy in treatment-naïve patients with systemic autoimmune diseases. Patients were classified into high-dose and low-dose glucocorticoid therapy groups, depending on their therapeutic regimen. We found that suppression of basal HPA axis function by glucocorticoid therapy in the low-dose group was closely related to inhibition of IL-6 production.

Cytokines may have a regulatory influence on the HPA axis [1–3]. For instance, IL-1 increases cortisol production by human adrenocortical cells as a non-ACTH regulator [9], but TNF-α decreases it [10]. With regard to regulation of the HPA axis by proinflammatory cytokines *in vivo*, administration of lipopolysaccharide (LPS) to mice has been reported to increase the ACTH level [11], and it was suggested that TNF-α, IL-1, and IL-6 are all involved in LPS-induced elevation of plasma ACTH. In the present study, 10 proinflammatory cytokines were measured over time in patients commencing glucocorticoid therapy. Among the 10 cytokines, only IL-6 showed a significant decrease from the second week of both high-dose and low-dose glucocorticoid therapy. We also found that basal cortisol level was positively correlated with IL-6 level before glucocorticoid treatment. Mastorakos *et al*. [12] reported that a week-long administration of recombinant IL-6 caused impressively marked and prolonged elevations of plasma ACTH and cortisol at the beginning of the study in human subjects, while with time it took over cortisol secretion granted that ACTH responses were diminished while cortisol responses remained strong. Thus, our finding of a decrease in IL-6 after the initiation of glucocorticoid therapy suggests that IL-6-induced cortisol production may be suppressed by glucocorticoids. It was also clear that IL-6 has the greatest impact on the HPA axis among the proinflammatory cytokines that we tested.

In addition, we found that the changes of IL-6 and basal cortisol during glucocorticoid therapy showed a significant positive correlation, while there was no significant correlation between the changes of basal ACTH and basal cortisol. These findings suggested that the reduction of basal cortisol was related to the decrease of IL-6, and was not influenced by ACTH. There were the separated values (the far left side in each figure) in Fig 4A, 4C and 4D. We therefore subanalyzed these relations without a separated value in these Figs. As a result, correlations of Fig 4A, 4C and 4D without a separate value showed a significant negative correlation (r = -0.4276, *P* = 0.0472), a significant positively correlation (r = 0.4681, *P* = 0.0280), and a trend of positive correlation (r = 0.3836, *P* = 0.0780), respectively. In an observational study of patients with PMR, Straub *et al*. found that an increase of serum IL-6 was associated with elevation of serum cortisol [13]. These results also suggest that IL-6 directly stimulates cortisol production. In addition, suppression of IL-6 by glucocorticoid therapy had a greater influence on the adrenal gland than the pituitary gland, since a positive correlation between ACTH and IL-6 was not observed in our patients. At present, the reason for this is not clear, although it is possible that suppression of basal ACTH production by the pituitary gland might have been caused by glucocorticoids or factors other than IL-6.

Cutolo *et al*. [14] reported that basal cortisol levels were decreased one month after starting low-dose glucocorticoid therapy in patients with PMR, which is similar to our findings. They conducted a CRH test just before starting glucocorticoid therapy, but they did not re-examine the CRH response after initiating therapy. Our study revealed that the response to CRH stimulation is maintained during at least 4 weeks of low-dose glucocorticoid therapy for inflammatory diseases, while basal cortisol levels are decreased.

The mean baseline IL-6 level was significantly higher in the low-dose group than in the high-dose group, possibly due to the different disease profiles of the two groups. PMR was the major disease in the low-dose group, and it generally has a high incidence in elderly persons and features marked systemic inflammation [15], while SLE [16] and PM/DM [17] were predominant in the high-dose group and are characterized by relatively less severe inflammation. We then subanalysed changes of serum proinflammatory cytokines by categorization of diseases in both high- and low-dose groups. Results in patients with PMR were almost the same as those in patient with other diseases in the low-dose group glucocorticoid therapy. Results in patients with SLE, PM/DM and SSc were also almost the same as those in patients with vasculitis syndrome in the high-dose group. Level of IL-6 at baseline in patients with SLE, PM/DM and SSc was lower than that of vasculitis syndrome, suggesting the different characteristics of inflammation.

There were also differences of the mean IL-10 and IL-12p70 levels between the high-dose and low-dose groups at baseline. Increased serum levels of IL-10 and IL-12 have been reported in SLE and PM/DM [18–20]. Because patients with SLE or PM/DM dominated the high-dose group, the disease profile might explain the differences of IL-10 and IL-12 between the two groups. However, IL-10 and IL-12p70 did not change significantly during glucocorticoid therapy.

Schuetz *et al*. reported that 89% of patients showed a blunted response to the ACTH test when it was performed after 2 weeks of prednisone therapy (40 mg daily) for acute exacerbation of chronic obstructive pulmonary disease [21]. In all of our patients from the high-dose group, stimulated serum cortisol levels were lower than 20 μg/ml after the CRH test, which was similar to the data of Schuetz.

In conclusion, at the initial phase of glucocorticoid therapy in patients with systemic autoimmune diseases, HPA axis function was markedly suppressed in the high dose group, but maintained albeit somewhat suppressed in the low-dose group. IL-6 was the only proinflammatory cytokine showing reduced levels during the initial phase of glucocorticoid therapy, and this change of IL-6 might contribute to the apparent suppression of the HPA axis.
